# Supplementary material for: The effects of genital myiasis on the diversity of the vaginal microbiota in female Bactrian camels
Source: BMC Vet Res. 2022 Mar 5;18:87. doi: 10.1186/s12917-022-03189-5 (PMC8897907; doi:10.1186/s12917-022-03189-5)
Supplement: Supplementary file 5 — Additional file 5. [file 12917_2022_3189_MOESM5_ESM.zip › MPL201709200_16s_yy/Treat1/css/js_css/jsPDF-master.zip!/jsPDF-master/docs/libs_css_colors.js.html]

libs/css\_colors.js - Documentation


## Home

### Classes

- FontObject
- jsPDF
- PubSub

### Global

- addFont
- addMetadata
- addPage
- CapJoinStyles
- circle
- CssColors
- ellipse
- getFontList
- html2pdf
- http
- lines
- lstext
- output
- rect
- requirejs
- reset
- roundedRect
- save
- setDisplayMode
- setDrawColor
- setFillColor
- setFont
- setFontSize
- setFontStyle
- setLineCap
- setLineJoin
- setLineWidth
- setPage
- setProperties
- setTextColor
- text
- toUrl
- triangle
- triggerEvent

# libs/css\_colors.js

```
/**
 * CssColors
 * Copyright (c) 2014 Steven Spungin (TwelveTone LLC)  steven@twelvetone.tv
 *
 * Licensed under the MIT License.
 * http://opensource.org/licenses/mit-license
 */

/**
 * Usage CssColors('red');
 * Returns RGB hex color with '#' prefix
 */

var CssColors = {};
CssColors._colorsTable = {
	"aliceblue" : "#f0f8ff",
	"antiquewhite" : "#faebd7",
	"aqua" : "#00ffff",
	"aquamarine" : "#7fffd4",
	"azure" : "#f0ffff",
	"beige" : "#f5f5dc",
	"bisque" : "#ffe4c4",
	"black" : "#000000",
	"blanchedalmond" : "#ffebcd",
	"blue" : "#0000ff",
	"blueviolet" : "#8a2be2",
	"brown" : "#a52a2a",
	"burlywood" : "#deb887",
	"cadetblue" : "#5f9ea0",
	"chartreuse" : "#7fff00",
	"chocolate" : "#d2691e",
	"coral" : "#ff7f50",
	"cornflowerblue" : "#6495ed",
	"cornsilk" : "#fff8dc",
	"crimson" : "#dc143c",
	"cyan" : "#00ffff",
	"darkblue" : "#00008b",
	"darkcyan" : "#008b8b",
	"darkgoldenrod" : "#b8860b",
	"darkgray" : "#a9a9a9",
	"darkgreen" : "#006400",
	"darkkhaki" : "#bdb76b",
	"darkmagenta" : "#8b008b",
	"darkolivegreen" : "#556b2f",
	"darkorange" : "#ff8c00",
	"darkorchid" : "#9932cc",
	"darkred" : "#8b0000",
	"darksalmon" : "#e9967a",
	"darkseagreen" : "#8fbc8f",
	"darkslateblue" : "#483d8b",
	"darkslategray" : "#2f4f4f",
	"darkturquoise" : "#00ced1",
	"darkviolet" : "#9400d3",
	"deeppink" : "#ff1493",
	"deepskyblue" : "#00bfff",
	"dimgray" : "#696969",
	"dodgerblue" : "#1e90ff",
	"firebrick" : "#b22222",
	"floralwhite" : "#fffaf0",
	"forestgreen" : "#228b22",
	"fuchsia" : "#ff00ff",
	"gainsboro" : "#dcdcdc",
	"ghostwhite" : "#f8f8ff",
	"gold" : "#ffd700",
	"goldenrod" : "#daa520",
	"gray" : "#808080",
	"green" : "#008000",
	"greenyellow" : "#adff2f",
	"honeydew" : "#f0fff0",
	"hotpink" : "#ff69b4",
	"indianred " : "#cd5c5c",
	"indigo" : "#4b0082",
	"ivory" : "#fffff0",
	"khaki" : "#f0e68c",
	"lavender" : "#e6e6fa",
	"lavenderblush" : "#fff0f5",
	"lawngreen" : "#7cfc00",
	"lemonchiffon" : "#fffacd",
	"lightblue" : "#add8e6",
	"lightcoral" : "#f08080",
	"lightcyan" : "#e0ffff",
	"lightgoldenrodyellow" : "#fafad2",
	"lightgrey" : "#d3d3d3",
	"lightgreen" : "#90ee90",
	"lightpink" : "#ffb6c1",
	"lightsalmon" : "#ffa07a",
	"lightseagreen" : "#20b2aa",
	"lightskyblue" : "#87cefa",
	"lightslategray" : "#778899",
	"lightsteelblue" : "#b0c4de",
	"lightyellow" : "#ffffe0",
	"lime" : "#00ff00",
	"limegreen" : "#32cd32",
	"linen" : "#faf0e6",
	"magenta" : "#ff00ff",
	"maroon" : "#800000",
	"mediumaquamarine" : "#66cdaa",
	"mediumblue" : "#0000cd",
	"mediumorchid" : "#ba55d3",
	"mediumpurple" : "#9370d8",
	"mediumseagreen" : "#3cb371",
	"mediumslateblue" : "#7b68ee",
	"mediumspringgreen" : "#00fa9a",
	"mediumturquoise" : "#48d1cc",
	"mediumvioletred" : "#c71585",
	"midnightblue" : "#191970",
	"mintcream" : "#f5fffa",
	"mistyrose" : "#ffe4e1",
	"moccasin" : "#ffe4b5",
	"navajowhite" : "#ffdead",
	"navy" : "#000080",
	"oldlace" : "#fdf5e6",
	"olive" : "#808000",
	"olivedrab" : "#6b8e23",
	"orange" : "#ffa500",
	"orangered" : "#ff4500",
	"orchid" : "#da70d6",
	"palegoldenrod" : "#eee8aa",
	"palegreen" : "#98fb98",
	"paleturquoise" : "#afeeee",
	"palevioletred" : "#d87093",
	"papayawhip" : "#ffefd5",
	"peachpuff" : "#ffdab9",
	"peru" : "#cd853f",
	"pink" : "#ffc0cb",
	"plum" : "#dda0dd",
	"powderblue" : "#b0e0e6",
	"purple" : "#800080",
	"red" : "#ff0000",
	"rosybrown" : "#bc8f8f",
	"royalblue" : "#4169e1",
	"saddlebrown" : "#8b4513",
	"salmon" : "#fa8072",
	"sandybrown" : "#f4a460",
	"seagreen" : "#2e8b57",
	"seashell" : "#fff5ee",
	"sienna" : "#a0522d",
	"silver" : "#c0c0c0",
	"skyblue" : "#87ceeb",
	"slateblue" : "#6a5acd",
	"slategray" : "#708090",
	"snow" : "#fffafa",
	"springgreen" : "#00ff7f",
	"steelblue" : "#4682b4",
	"tan" : "#d2b48c",
	"teal" : "#008080",
	"thistle" : "#d8bfd8",
	"tomato" : "#ff6347",
	"turquoise" : "#40e0d0",
	"violet" : "#ee82ee",
	"wheat" : "#f5deb3",
	"white" : "#ffffff",
	"whitesmoke" : "#f5f5f5",
	"yellow" : "#ffff00",
	"yellowgreen" : "#9acd32"
};

CssColors.colorNameToHex = function(color) {
	color = color.toLowerCase();
	if (typeof this._colorsTable[color] != 'undefined')
		return this._colorsTable[color];

	return false;
};
```

  

Documentation generated by JSDoc 3.4.2 on Sat Oct 08 2016 21:42:26 GMT+0100 (BST) using the docdash theme.
